# How Can Plant-Derived Natural Products and Plant Biotechnology Help Against Emerging Viruses?

**DOI:** 10.3390/ijms26157046

**Published:** 2025-07-22

**Authors:** Gergana Zahmanova, Katerina Takova, Valeria Tonova, Ivan Minkov, Momchil Barbolov, Neda Nedeva, Deyana Vankova, Diana Ivanova, Yoana Kiselova-Kaneva, Georgi L. Lukov

**Affiliations:** 1 Department of Molecular Biology, University of Plovdiv, 4000 Plovdiv, Bulgaria; 2 Center of Plant Systems Biology and Biotechnology, 4000 Plovdiv, Bulgaria; 3 Institute of Molecular Biology and Biotechnologies, 4108 Markovo, Bulgaria; 4 Department of Biochemistry, Molecular Medicine and Nutrigenomics, Medical University Varna, 9002 Varna, Bulgaria; 5 Faculty of Sciences, Brigham Young University–Hawaii, Laie, HI 96762, USA; georgi.lukov@byuh.edu

**Keywords:** medicinal plant, phytotherapy, phytochemicals, viral infection, plant-derived antivirals, vaccines, antibodies, plant molecular farming

## Abstract

Infectious diseases have been treated using plants and their compounds for thousands of years. This knowledge has enabled modern techniques to identify specific antiviral remedies and to understand their molecular mechanism of action. Numerous active phytochemicals, such as alkaloids, terpenoids, polyphenols (phenolic acids, flavonoids, stilbenes, and lignans), coumarins, thiophenes, saponins, furyl compounds, small proteins, and peptides, are promising options for treating and preventing viral infections. It has been shown that plant-derived products can prevent or inhibit viral entry into and replication by host cells. Biotechnological advances have made it possible to engineer plants with an increased capacity for the production and accumulation of natural antiviral compounds. Plants can also be engineered to produce various types of antivirals (cytokines, antibodies, vaccines, and lectins). This study summarizes the current understanding of the antiviral activity of specific plant-derived metabolites, emphasizing their mechanisms of action and exploring the enormous potential of plants as biological factories.

## 1. Introduction

Emerging viral infections continue to be a significant global health concern. Phytochemicals with antiviral properties, together with advances in plant molecular farming, position plants as both sources of therapeutic compounds and platforms for producing valuable pharmaceutical proteins for viral prevention and treatment. Here, we highlight the potential of plant-based approaches in addressing current and future viral threats.

### 1.1. The Persisting Era of Infectious Disease

**Figure 1:**
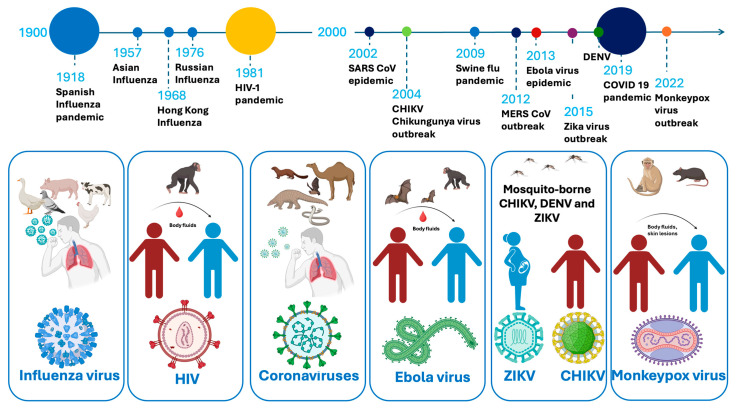
A timeline of viral epidemics and pandemics from 1900 to the present, the viral host, viral vectors, and transmission methods.

A substantial number of severe illnesses are caused by emerging and reemerging viruses, which have become increasingly prevalent due to factors such as population growth, global travel, and climate change [1,2,3]. Over the past 40 years, multiple major outbreaks have demonstrated the unpredic and evolving nature of viral epidemics. These include the 1981 human immunodeficiency virus-1 (HIV-1) pandemic, the 2002 Severe Acute Respiratory Syndrome coronavirus (SARS-CoV) outbreak, and the 2009 influenza A H1N1 (swine flu) pandemic. In 2012, the Middle East Respiratory Syndrome coronavirus (MERS-CoV) emerged, followed by the 2013 Ebola virus (EBOV) epidemic in West Africa. The 2015 Zika virus (ZIKV) outbreak in the Americas and the 2019 dengue fever virus (DENV) epidemic further underscored the global vulnerability to arboviruses. The emergence of SARS-CoV-2 in 2019 rapidly escalated into a worldwide pandemic. More recently, the 2022 Monkeypox virus (MPXV) outbreak and the 2024 resurgence of the chikungunya virus (CHIKV) in La Réunion, following its major outbreak in 2004, emphasize the ongoing challenge posed by viral epidemics (Figure 1) [4].

However, the mortality and morbidity associated with infectious diseases have decreased over the past forty years due to the swift development of vaccines, improvements in sanitation, access to healthcare, and medical advancements [5]. New and improved biotechnologies, such as the development of mRNA vaccines and recombinant virus-like particles (VLPs) as vaccine and delivery vesicles, and the use of new expression systems (mammalian, insect, and plant cells, and whole plants), offer new approaches for rapid vaccine preparation, especially against the emergence of new viruses [6,7]. Controlling the spread of (re)emerging viruses may also depend on using robust antivirals. Traditional plant-derived remedies and plant biotechnology products can be a source of affordable new antiviral agents for anti-inflammatory and immunomodulatory therapy that can support the battle against (re)emerging viruses [8,9].

### 1.2. Antivirals

**Figure 2:**
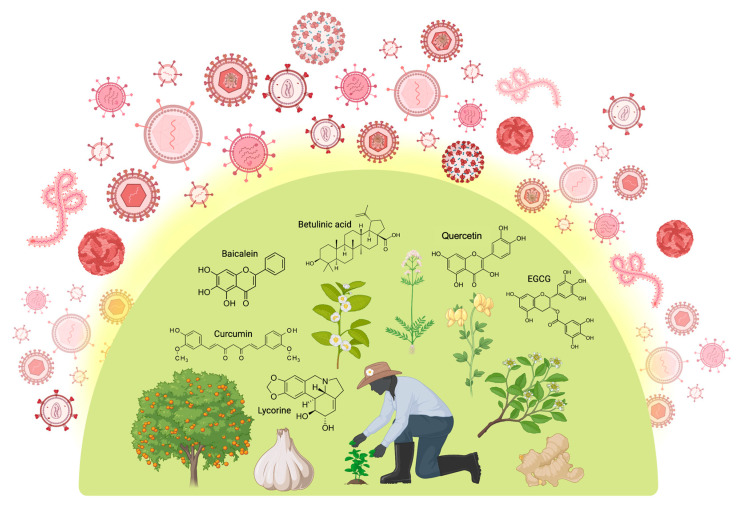
Medicinal plants and plant-derived phytochemicals offer broad-spectrum protection against various stages of the viral life cycle. Polyphenols (e.g., quercetin, baicalein, curcumin, EGCG), followed by terpenoids and alkaloids such as betulinic acid and lycorine, respectively, are the most commonly occurring structural families of phytochemicals bearing pronounced antiviral properties. Created in BioRender [14].

Antiviral drugs, natural and synthetic, are used to treat viral infections, with the latter synthesized like traditional medications [10]. The abundance of bioactive agents with therapeutic qualities in plants makes them suitable for use in medicine. The primary source of remedies in almost 80% of developing nations is herbal [11], and over 40% of medications today come from natural products and traditional knowledge [12]. Plant compounds such as alkaloids, flavonoids, polyphenols, and tannins have been studied for their potential antiviral properties, and their effectiveness is being evaluated continuously [13]. Figure 2 presents an overview of medicinal plants and plant-derived phytochemicals that have been used for viral protection.

Plant biotechnology can also advance antiviral treatments, from improving the production of natural antiviral compounds to aiding in the development of plant-based vaccines, antibodies, and therapeutic and diagnostic proteins. By harnessing the unique properties of plants and advancing genetic engineering, biotechnology has the potential to offer sustainable solutions for both human and animal health.

This review highlights the significant role of plant-derived compounds and plant biotechnology in antiviral research, focusing on recent advancements in the field. An alternative strategy for creating safe, efficient, and cost-effective antivirals and vaccines is the use of recombinant proteins and small molecules expressed in plants. By harnessing the natural capabilities of plants, we can produce therapeutic compounds, offering a sustainable and scalable approach for combating viral infections.

## 2. Life Cycle of (Re)Emerging Viruses—How Can Natural Products Help?

(Re)emerging viruses continue to pose significant threats to global health, with their ability to rapidly mutate, adapt to new environments, and spread across populations [15]. The increasing frequency of viral outbreaks (Figure 1) underscores the urgent need to improve the production of plant remedies, plant-derived therapeutics, and vaccines. However, identifying herbal or herbal-based remedies or developing synthetic agents that can suppress or disrupt the life cycle of viruses without causing harm to the host’s cells is a notable challenge. Furthermore, viruses utilize host cellular machinery for the synthesis and assembly of their basic components—nucleic acid (DNA or RNA), the protein coat, viral enzymes, and, occasionally, a lipid envelope—which significantly complicates the development of safe-for-the-host antivirals [11,16].

**Figure 3:**
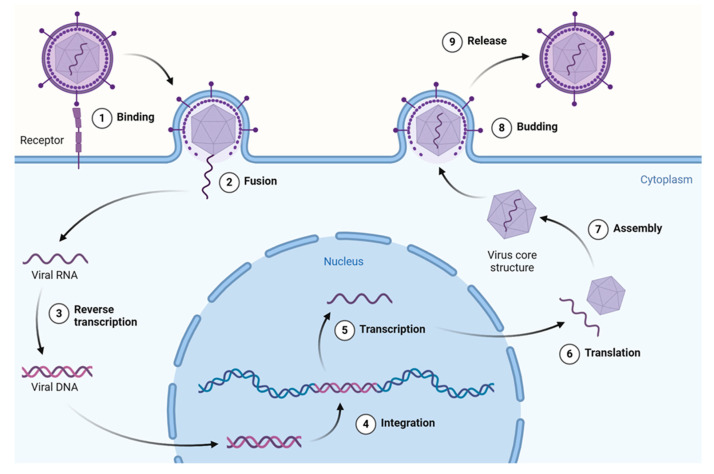
Overview of the retrovirus (HIV-1) life cycle. This figure illustrates key stages from viral entry and reverse transcription to integration, replication, assembly, and budding of the mature virion. HIV-1 is shown here as a model system due to its extensively studied replication pathway and the availability of data on plant-derived compounds targeting various stages. Created using a template retrieved from Goldman-Israelow. Created in BioRender [14].

In general, the viral life cycle comprises the following events—attachment, penetration, uncoating, transcription, replication, assembly, and release—with most antivirals targeting at least one of the above steps. This cycle repeats multiple times, leading to viral spread within the host organism and the onset of potentially pathological conditions. The stages of the retrovirus life cycle are described in Figure 3.

Current antiviral therapeutics exhibit their properties either by inhibiting virus attachment or entry into the cell, increasing the cell’s resistance to the virus, or deproteinizing the virus, or by producing antimetabolites that inhibit nucleic acid synthesis.

## 3. Plant Compounds That Target Stages of the Viral Life Cycle

A common feature of all viruses is the exploitation of host proteins for their efficient entry, replication, assembly, and exit from infected cells, all while trying to evade the host immune system. This is achieved through mechanisms of prioritizing viral protein expression and specific interactions between viral and host proteomes. Plant-derived preparations have been used to treat infectious diseases throughout human history. Phytochemicals have been shown to impact the different stages of the viral life cycle by interacting with specific viral proteins and reducing viral infectivity. Below, we describe proteins involved in coronavirus, HIV-1, influenza, dengue, Zika, Ebola, and chikungunya virus entry, replication, and viral protein synthesis and maturation. Plant-derived bioactive compounds that have been proven to exhibit antiviral properties through in vitro experiments are listed in Appendix A (Table A1 and Table A2), along with their respective specific viral protein targets and calculated IC_50_/EC_50_ values.

### 3.1. Phytochemicals Modulating Viral Entry, Attachment, and Fusion

Viruses must first enter their target cells through interactions between external viral structural proteins and host cell surface proteins, which trigger receptor-mediated endocytosis or direct membrane fusion.

Coronaviruses (CoVs) are a family of enveloped, positive-sense, single-stranded RNA viruses. Seven species of CoVs are pathogenic for humans, and three of them have caused pandemics of severe disease—the SARS-CoV and MERS-CoV outbreaks in 2002 and 2012, respectively, and SARS-CoV-2, which emerged in Wuhan, China, in December 2019 and caused the COVID-19 pandemic, which spread rapidly across the world due to the high transmissibility and pathogenicity of the virus [17,18,19,20]. The high pathogenicity and mortality of COVID-19 revealed the urgent need for broad-spectrum antivirals to meet the challenges of current and future coronavirus outbreaks [21].

Coronaviruses utilize the spike (S) proteins on their membrane envelopes to bind cell surface proteins such as angiotensin-converting enzyme 2 (ACE-2; SARS-CoV and SARS-CoV-2) or dipeptidyl peptidase 4 (DPP4; MERS-CoV) via the S1 subunit, while the S2 subunit mediates membrane fusion following cleavage by host surface proteases like transmembrane protease serine 2 (TMPRSS2), cathepsins, and furin [22]. Therefore, both the interaction between S proteins and their host surface protein targets and the cleavage of the S protein necessary for fusion can be targeted by antivirals.

Several molecules of plant origin have been identified by in vitro experiments as potent inhibitors of the processes of interaction and fusion of CoVs, such as cepharantine, neferine, hernandezine, and various agglutinin lectins [23,24]. Punicalin and punicalagin found in *Gunnera perpensa* inhibited the process of viral entry by the disruption of spike glycoprotein–host ACE2 interaction at very low concentrations—IC_50_ 0.009 µM and 0.029 µM, respectively [25]. Similarly, quinoline carboxylic acids present in *Ephedra sinica* also blocked the interaction between the SARS-CoV-2 receptor-binding domain and ACE2 in a dose-dependent manner and with a low IC_50_ concentration [26]. The anthraquinone emodin, found in various plants, has been shown to block S protein binding to ACE2 in vitro and to limit SARS-CoV infectivity in Vero E6 cells [27]. Ohishi and coauthors tested 10 phytochemicals, and epigallocatechin gallate (the major flavonoid in green tea) exhibited the strongest inhibition of S protein–ACE2 binding, as confirmed by ELISA experiments [28]. Other phytochemicals have been proven to impair the cleavage of the S protein by host proteases and, thus, prevent virus fusion. The large polyphenol tannic acid inhibited host TMPRSS2-mediated proteolysis of the S protein with an IC_50_ of 2.31 µM, as well as the main viral 3CL protease, as discussed later [29]. The ubiquitous flavonol quercetin, found in many fruits and vegetables, exhibited the blockage of furin-mediated S protein cleavage [30].

The entry of human immunodeficiency virus (HIV), a retrovirus (+ssRNA), is mediated by the interaction between gp120 and gp41 envelope glycoprotein heterodimers and the CD4 T-cell surface protein, along with the engagement of the chemokine receptor CCR5 or CXCR4. The multistep interaction triggers conformational changes in gp41, which facilitate the fusion of the viral envelope with the host membrane [31]. Abrogating the binding between gp120 and CD4 or gp41-mediated membrane fusion is therefore a viable antiviral strategy.

Disrupted HIV-1 entry by the targeting of gp120, gp41, or the necessary host cell receptors has been reported for several phytochemicals. Baicalin, a flavonoid from *Scutellaria baicalensis*, has been widely studied in regard to its inhibitory potential, including demonstrated effects against HIV-1 entry by binding to gp120 [32].

The host cell entry of the mosquito-borne *Flaviviridae* (+ssRNA) family members, the dengue (DENV) and Zika (ZIKV) viruses, is cell-type-specific and is mediated through the major structural envelope (E) viral glycoprotein binding to various host factors such as surface glucosaminoglycans, the phosphatidylserine receptor TIM-1, C-type lectins such as DC-SIGN and L-SIGN, and others. These interactions trigger clathrin-mediated endocytosis, and the subsequent acidification of the endosomes causes the formation of fusogenic E glycoprotein trimers, which facilitate fusion between the viral envelope and the endosomal membranes, resulting in the release of viral RNA into the cytosol [33].

Various phytochemicals have been shown to interfere with the entry of DENV and ZIKV by targeting either the viral E protein or crucial host entry factors. For example, naringenin, a flavonoid found in citrus fruits, has been shown to inhibit DENV infection in Huh7.5 cells [34]. Similarly, baicalein demonstrated potent inhibitory effects on DENV-2 infection in human liver cells by blocking viral adsorption, and baicalin inhibited viral attachment to host cells by binding ZIKV envelope (E) protein [35,36,37]. Gossypol has been found to be an effective inhibitor of both DENV and ZIKV envelope protein region III attachment [38]. In the context of ZIKV, EGCG again emerges as a promising entry inhibitor due to its direct binding to the ZIKV envelope protein, preventing viral adsorption and internalization into host cells [39]. Curcumin has been experimentally validated to inhibit ZIKV infection by impairing viral binding to host cells and suppressing membrane fusion, through the destabilization of the viral envelope [40].

Furthermore, several studies highlight compounds capable of affecting post-entry processes linked to viral protein translation and replication. Isoquercitrin, another flavonoid, was shown to inhibit the internalization of ZIKV particles into host cells without affecting initial binding, suggesting an impact on endosomal maturation or membrane fusion steps [41]. Unlike HIV, which uses receptor-mediated conformational changes, or coronaviruses, which require proteolytic priming, flavivirus entry depends critically on low-pH-triggered E protein rearrangements, offering unique intervention points for antiviral phytochemicals.

The arthritogenic chikungunya virus (CHIKV) is a mosquito-borne alphavirus, a member of the *Togaviridae* family (+ssRNA). It enters target mammalian cells through receptor-mediated endocytosis via the interaction of its envelope glycoprotein with several possible cell surface receptors—matrix remodeling-associated protein 8 (MXRA8), prohibitin 1, and the TIM1 phosphatidylserine receptor—potentially also aided by binding to glucosaminoglycans such as heparan sulfate [42,43,44,45]. After the acidification of the endocytic compartment, conformational changes in the E1 viral envelope glycoprotein trigger fusion with the endosomal membrane, allowing the nucleocapsid to enter the cytosol and initiate infection.

The interaction between CHIKV E1 glycoprotein and host factors and its subsequent entry have been shown to be abrogated by some of the phytochemicals commonly mentioned in this article—epigallocatechin gallate, baicalein, and curcumin as well as the antimalarial chloroquine [40,46,47,48]. Flavaglines FL3 and FL23, along with sulfonyl amidine, also have an inhibitory effect on entry via binding to prohibitin, while the *Tectona grandis* (Teak) compounds 2-(butoxycarbonyl) benzoic acid (BCB), 3,7,11,15-tetramethyl-1-hexadecanol (THD), and benzene-1-carboxylic acid-2-hexadeconate (BHCD) exert their inhibition by directly binding the E1 CHIKV envelope glycoprotein [49,50].

Influenza viruses, belonging to the Orthomyxoviridae family (-ssRNA), initiate infection by utilizing the binding of their trimeric surface glycoprotein hemagglutinin (HA) to sialic acid residues on host surface glycoproteins and glycolipids. The virus is then internalized via clathrin-mediated endocytosis, and within the acidic environment of endosomes, HA undergoes a conformational change which promotes the fusion of the viral and endosomal membranes, facilitating the release of viral ribonucleoprotein complexes (vRNPs) into the cytoplasm and their subsequent transport into the nucleus for replication [51]. The additional steps of pH-dependent fusion, endosomal trafficking, and nuclear import, absent from the life cycles of the aforementioned viruses, provide further intervention points for potential antiviral strategies. Several phytochemicals have been validated to interfere with influenza virus entry, predominantly by targeting HA-mediated binding or fusion processes.

EGCG and other catechins from green tea have been shown to inactivate influenza virions by binding to HA and blocking attachment to host cells, with experimental confirmation in vivo [52]. Quercetin, a ubiquitous flavonol found in many fruits and vegetables, and its derivatives have also been reported to inhibit influenza virus infection by the same mechanism [53,54]. Moreover, some compounds target host factors that influenza viruses exploit during entry. For instance, resveratrol, a stilbenoid from *Vitis vinifera* (grapes), has been found to inhibit nuclear–cytoplasmic transport of viral ribonucleoproteins by modulating host PI3K/Akt signaling, thereby impairing an essential post-entry step in the influenza replication cycle [55]. Several alkaloids (harmalol, harmane, harmaline, and strychnine sulfate) had an anti-IVA adsorption effect in MDCK cells [56].

Ebola virus (EBOV), a member of the Filoviridae family (-ssRNA), initiates infection through the engagement of its heavily glycosylated surface glycoprotein (GP) with multiple host cell binding factors such as C-type lectins (e.g., DC-SIGN, L-SIGN) and phosphatidylserine receptors like TIM-1 [57]. Following internalization through macropinocytosis, EBOV traffics to late endosomes, where its GP is cleaved by endosomal proteases, notably cathepsins B and L, thus exposing the receptor-binding site necessary for interaction with the resident endosomal Niemann–Pick C1 membrane protein (NPC1). Binding to NPC1 facilitates the fusion of the viral and endosomal membranes, enabling the release of the nucleocapsid into the cytoplasm, initiating replication [58]. The reliance on host late endosomal proteases and the additional interaction with the NPC1 protein provide further points of intervention for small-molecule antivirals.

The targeting of GP-mediated binding has been demonstrated for several proanthocyanidins and flavanols from the *Maesa perlarius* plant, with procyanidin b2 demonstrating the strongest EBOV GP binding [59]. Additionally, quercetin in its glycosidic form has demonstrated inhibitory effects on EBOV infection by impairing viral entry, likely through interactions with GP or the disruption of endosomal trafficking, as shown in Vero E6 cell culture [60].

### 3.2. Phytochemicals Modulating Viral Replication, Protein Synthesis, and Maturation

Following entry into the host cell, viruses must rapidly and efficiently replicate their genomes and produce structural and non-structural proteins using host biosynthetic machinery, through interactions between viral and host proteins, allowing viruses to hijack or bypass cellular systems such as their transcriptional machinery, RNA processing pathways, and ribosomes. Viral protein production is prioritized over host proteins through various mechanisms including co-opting host RNA polymerases, employing RNA cap-snatching, generating internal ribosome entry sites (IRESs), or selectively modulating which mRNAs are translated [61]. These mechanisms not only ensure the production of essential viral components but can also serve to suppress host antiviral responses. The disruption of the interactions between viral and host proteins at this stage is also an attractive avenue for the development of antivirals.

CoV positive-sense mRNA is directly translated by host ribosomes into the pp1a and pp1ab polyproteins upon entry into the cytosol. They are then autocatalytically cleaved by the action of the viral main protease (M^pro^/3CL^pro^, nsp5) and papain-like protease (PL^pro^, nsp3) within the polyprotein polypeptide chains, resulting in the generation of several non-structural proteins (NSPs), which facilitate selective translation and the formation of the replication–transcription complex (RTC), sequestered within endoplasmic reticulum-derived double-membrane vesicles. The viral RNA-dependent RNA polymerase (RdRp, nsp12) then produces full-length genomic RNA and nested subgenomic RNAs through discontinuous transcription, which yield the main structural viral proteins (S, E, M, and N). RNA helicase (nsp13) is a nucleoside triphosphatase (NTPase). Once viral proteins are synthesized, nucleocapsids are assembled in the cytoplasm and budded into the lumen of the endoplasmic reticulum (ER)–Golgi intermediate compartments. Virions are then released from the infected cell through exocytosis [62,63,64,65]. SARS-CoV-2 genome characterization provides the basis for further studies on pathogenesis and the optimization of the design of diagnostic, antiviral, and vaccination strategies [66].

Many phytochemicals have been shown in recent years to target specific proteins involved in virus replication and maturation, both in vitro and in vivo. For example, baicalin and baicalein, main flavonoids found in Scutellaria baicalensis, inhibit SARS-CoV-2 3CLPro and SARS-CoV MPro proteinases as demonstrated by isothermal titration calorimetry and enzyme binding FRET assays and in vitro assays, respectively [67,68,69]. Baicalein also inhibited SARS-CoV-helicase unwinding and ATP-ase activity [70]. The same study also examined 21 compounds of plant origin, and myricetin, quercetin, flavanone, kaempferol, and licoflavone C were found to be efficient unwinding activity inhibitors as well. Quercetin and kaempferol and their derivatives are among the most ubiquitous polyphenols in different plant parts [71]. Their aglycones and glycosides are also potent SARS-CoV 3CLPro, SARS-CoV PLPro, SARS-CoV-2 MPro, and SARS-CoV-helicase inhibitors [70,72,73,74]. Amentoflavone is a biflavonoid of apigenin and is present in a variety of plants, such as *Ginkgo biloba* and *Hypericum perforatum* [75,76]. Amentoflavone, apigenin, and its glycoside were established to inhibit SARS-CoV 3CLPro, SARS-CoV-2 RdRp, and SARS-CoV helicase in vitro [70,73,77,78]. Catechins are present in high quantities in different *Camellia sinensis* herbal remedies [79]. Several studies established that different catechin representatives exert inhibitory activity on proteins, responsible for CoV replication [70,80,81,82,83]. FRET analysis revealed cyanidin 3-O-galactoside to be a potent SARS-CoV-2 MPro inhibitor [82]. This compound is present in high concentrations in *Vaccinium* species, *Aronia melanocarpa*, and *Sambucus ebulus* [84,85,86]. A strong MERS-CoV RdRp inhibitor is lycorine, found in *Lycoris radiata* [87]. CoV RdRP is highly conserved among coronaviruses, and its low mutation rate makes it a promising target for drug development [88,89,90].

The HIV genome is composed of two identical single-stranded RNA molecules. The genome of the HIV provirus, also known as proviral DNA, is generated during reverse transcription by reverse transcriptase (RT) of the viral RNA genome into DNA, the degradation of the RNA, and the integration of the double-stranded HIV DNA into the human genome by HIV-1 integrase [91]. HIV-1 RT inhibition by chicoric acid and 1-methoxyoxalyl-3,5-dicaffeoylquinic acid was demonstrated by McDougall and coauthors [92]. Geraniin and corilagin have also been shown to be effective RT inhibitors in vitro [93]. In a study from 2021, anti-HIV-1 integrase and anti RT-associated RNAse activities were demonstrated in vitro for 11 compounds, isolated from *Punica granatum* [94]. Among them, apigenin, ellagic acid, luteolin, luteolin 7-O-glucoside, punicalins, and punicalagins were the most potent inhibitors of both enzymes. Strong inhibition of RT-associated RNA-se was detected for betulinic, oleanolic, and ursolic acids as well. A derivative of betulinic acid (3-O-(3′,3′-dimethylsuccinyl) betulinic acid) decreased RT-associated RNAse in cell culture [95].

The ZIKV genomic RNA encodes a large polyprotein that is co- and post-translationally processed into structural capsid (C), pre-membrane (prM), and envelope (E) proteins, and non-structural proteins (NS1, NS2a, NS2b, NS3, NS4A, NS4B, and NS5) [96]. The main biological role of NS5, the largest non-structural ZIKV protein, is the synthesis and capping of the viral RNA. It consists of two major functional domains—the N-terminal methyltransferase (MTase) and the C-terminal RNA-dependent RNA polymerase (RdRP) [97,98]. However, to date, effective preventive measures against the Zika disease are still lacking [99,100]. Enzyme interaction analysis established catechins, luteolin, and myricetin as possible efficient pZIKV NS2B-NS3Pro inhibitors [101]. ZIKV helicase activity is impaired by EGCG, and ZIKV RdRp activity is impaired by lycorine [102,103].

The dengue virus (DENV) genome encodes three structural proteins (C, M, and E), which form part of the mature virion, and seven non-structural proteins (NS1, NS2A, NS2B, NS3, NS4A, NS4B, and NS5) [104]. NS5 is involved in mRNA capping through its methyltransferase and guanylyltransferase activities [105]. Attempts to develop anti-DENV antiviral agents appear to be very challenging due to the presence of four dengue serotypes (DENV1, DENV2, DENV3, and DENV4) that are highly prone to mutation due to the error-prone nature of their RNA polymerase [106]. Several studies focus on the few anti-DENV plant compounds that can interact with key viral proteins in vitro and in cell culture. Sotetsuflavone and apigenin are DENV RdRp inhibitors [107,108]. Myricetin is a potent DENV NS1 protease inhibitor, while bisdemethoxycurcumin and curcumin are DENV NS2B/NS3 protease inhibitors [109,110].

Chikungunya virus (CHIKV) has a ~12 kb positive-sense RNA genome with two open reading frames—the 5′-proximal ORF encodes non-structural replication proteins nsp1-4, while the 3′-ORF encodes the structural capsid, E1-3, and 6K proteins [111,112]. Upon exit from the endocytic compartment, the uncoated viral RNA is directly translated into one of the non-structural polyproteins nsp1-4, which then undergoes autoproteolysis by virtue of the nsp2 protease/helicase domain. The replication complex is then assembled and the RNA-dependent RNA polymerase nsp4 produces a negative-sense RNA template for genomic RNA synthesis and for the production of subgenomic RNA, encoding the structural polyprotein, which is then further cleaved. Other than the phytochemicals blocking its entry, several studies have found inhibitory effects of natural compounds on CHIKV replication. One study identified apigenin (IC_50_ = 70.8 µM), chrysin (IC_50_ = 126.6 µM), narigenin (IC_50_ = 118.4 µM), and silybin (IC_50_ = 92.3 µM) as potent inhibitors of CHIKV replication in BHK-21 cells, while harringtonine from *Cephalotaxus harringtonia* lowers negative- and positive-sense RNA and viral protein production in the same cell line [113,114]. Among other phytochemicals showing promising reductions in CHIKV viral replication or protein synthesis are berberine, prostratin, baicalein, and silymarin [48,115,116,117].

The genome of influenza A virus (IAV) is divided into eight segments—viral RNAs (vRNAs)—all present in a single IAV virion, that encode at least 16 different viral proteins, using partially overlapping open reading frames and alternative splicing [118,119]. The vRNAs are single-stranded, except for the last 13–14 nucleotides of the 5′ and 3′ termini, which are partially complementary [120]. Influenza neuraminidase (NA) cleaves the glycosidic linkage of terminal sialic acid residues on host glycoproteins. It plays a role in the release of progeny virions from infected cells [121,122]. Due to its important role in the viral life cycle, NA is considered the primary target for influenza antivirals. Several compounds have been tested for anti-IVA NA activity. Among them, gossypetin, herbacetin, kaempferol, and quercetin showed very low IC_50_ concentrations [123]. Other studies have revealed hispidulin, luteolin, matteucin, matteucinol, methoxymatteucin, and nepetin to also have the same activity [124,125]. 2′,4′dihydroxy-6′-methoxy-3′,5′-dimethylchalcone and myricetin-3′,5′-dimethylether-3-O-β-D-galactopyranoside exhibited inhibition against NA of four IVA subtypes in HEK293 cell culture and oxypeucedanin against two IVA subtypes [126,127]. IVA RdRp in vitro inhibition was also established for catechins [128]. Berberine, an active ingredient of *Berberis* sp., interfered with IVA maturation by blocking the transport of influenza A ribonucleoprotein to the cytoplasm [129]. IVA is also known to upregulate the mitogen-activated protein kinase/extracellular signal-related kinase (MAPK/ERK) pathway and hijacks this pathway for nucleolar export of the viral ribonucleoprotein for virus maturation, with berberine hampering this interaction [129].

Ebola virus (EBOV) is composed of seven genes coding at least ten proteins, among which are the following: nucleoprotein (NP), viral protein 35 (VP35), VP40, glycoprotein (GP), soluble GP (sGP), Δ-peptide, ssGP, VP30, VP24, and polymerase (L). VP35 is a component of the EBOV polymerase complex, where it serves as a bridge between the viral RNA-dependent RNA polymerase (L) and the nucleoprotein (NP) [130,131]. It is also involved in viral genome packaging and nucleocapsid formation and has been reported to possess NTPase- and helicase-like activities potentially involved in viral RNA remodeling [132,133]. Several plant components have been tested for VP35–dsRNA interactions, where cynarin and germacrane sesquiterpene 8α-(5′-hydroxyangeloyl)-salonitenolide were identified as the most potent with 90% and 60% inhibitory activity, respectively, at 100 µM [134].

## 4. (Re)Emerging Viruses—How Can Plant Biotechnology Help?

The use of engineered plants to produce high-quality molecules (vaccines, antibodies, cytokines, peptides, and bioactive small molecules) with pharmaceutical applications is increasing [135]. This area of biotechnology is also known as plant molecular farming (PMF) [136,137]. The advancement and optimization of PMF are due to the successful application of well-established techniques, such as plant gene engineering, increasing yields through alternative expression methods using plant viruses, genome editing biosynthetic pathways, modeling plant glycoengineering, and improving downstream processing (DSP) [138,139,140,141].

### 4.1. Plant-Derived Vaccines

**Figure 4:**
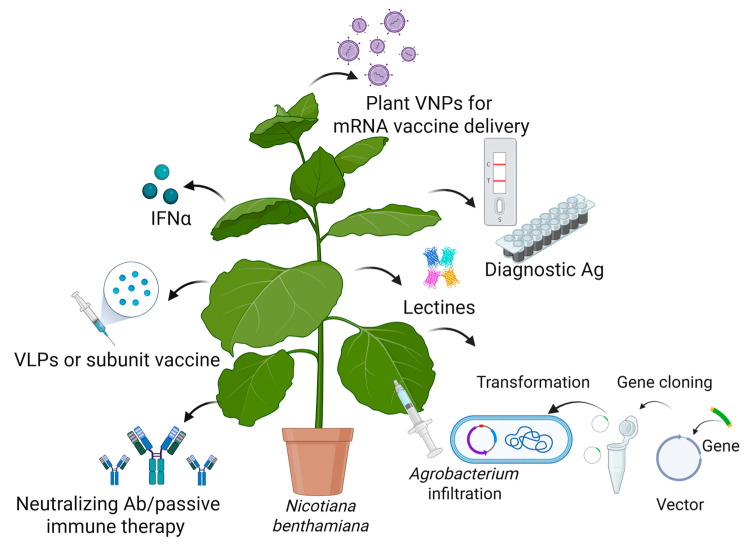
Transient expression of recombinant proteins (antigens (Ag), antibodies (Ab), viral nanoparticles (VNPs), vaccines, virus-like particles (VLPs), lectins, interferon α (INFα)) in *N. benthamiana* and their application in the fight against emerging viruses. Created in BioRender [14].

Plant biotechnology and vaccine production have emerged as a promising field that can address some of the global challenges posed by (re)emerging viruses. Plant-derived vaccines are produced using recombinant biotechnology, where the gene encoding the desired antigen is inserted into a viral vector or integrated into the plant genome [142,143]. Effective vaccinogens can then be cost-effectively manufactured in plants, providing a massive number of doses for global-scale deployment in a relatively short amount of time. PMF is based on three main technological platforms: transient expression in *Nicotiana benthamiana* using viral vectors, stable transgenic or transplastomic plant production, and transgenic plant cell-suspension cultures [142,143,144,145,146]. Transient expression with agroinfiltration is the process of introducing genes into plant leaves by infiltrating them with binary vectors carried by *Agrobacterium tumefaciens.* Figure 4 describes the use of plants as biofactories to produce recombinant proteins with antiviral applications.

Considering the need for vaccines and therapeutics for rapidly spreading (re)emerging viruses, transient expression systems provide the necessary time efficiency and scalability [147,148]. During the COVID-19 pandemic, several companies (Medicago Inc., Québec, QC, Canada; Kentucky BioProcessing, Owensboro, KY, USA; iBio, San Diego, CA, USA; Baiya Phytopharm, Bangkok, Thailand) used *N. benthamiana* to develop a vaccine against SARS-CoV-2. Their candidate vaccines passed successful phase-1 and -2 clinical trials [148,149,150]. Medicago Inc.’s vaccine successfully passed phase-3 clinical trials, and in 2022, Health Canada approved the first plant-derived SARS-CoV-2 vaccine, Covifenz [151]. Using transient expression technology, Medicago Inc. also produced and licensed a vaccine against the influenza virus. Although stable plant transformation takes longer, this approach enables large-scale and long-term recombinant protein production. The stable transformation of edible plants (maize, rice, oilseeds, lettuce, alfalfa, and tomatoes) allows the development of orally delivered vaccines, especially for veterinary applications [6,152,153,154]. To identify the most efficient plant expression system (stable, transient, or transplantomic), the viral antigens are expressed both transiently and stably. For example, HIV-1 capsid protein p24 was expressed independently and fused with the matrix subunits p17 (p17/p24) using transient and stable expression in tobacco. The Agrobacterium-mediated transient expression of p24 and p17/p24 gave the highest yield (1 mg p24/kg fresh weight). The highest intracellular accumulation of p17/p24 was observed in the chloroplast compared to the endoplasmic reticulum (ER) and cytoplasm [155]. In conclusion, the optimal production method (stable or transient) and the site for achieving sustained and robust accumulation (cytosol, chloroplast, ER, or apoplast) need to be determined empirically based on the distinct features of the chosen immunogen. The route of vaccine delivery, the capacity to create high amounts of recombinant protein expression, and the low value of downstream processing all influence the selection of technology for plant-based vaccine expression.

Plant molecular farming and plant-derived vaccines offer workable solutions addressing some of the challenges caused by outbreaks of (re)emerging viruses [156,157,158,159]. The high cost of developing and producing new vaccines is a significant obstacle for the resource-limited countries that need them the most. Developing countries heavily depend on other regions for life-saving vaccines, 99% of which are imported [160]. This stark imbalance in vaccine production can lead to unequal access and significant health disparities between developing countries and other parts of the world. Plant biotechnology companies based in Africa or Asia, producing plant-derived vaccines, therapeutics, and diagnostic reagents, may solve production and supply problems locally. Companies like Cape Biologix Technologies, South Africa, Baiya Phytopharm, Thailand, and BioApplications Inc., South Korea, are good examples of the successful implementation of local products in local markets [149,161]. Plant-derived vaccines against (re)emerging viruses developed during the past 10 years are summarized in Table 1.

### 4.2. Bio-Encapsulation of mRNA Within Plant-Derived Virus-like Particles (VLPs) and Chimeric VLP Production

Virus-like particles (VLPs) that mimic a natural virion but lack the native genome have been used as vaccines or vehicles for drug and nucleic acid delivery [168]. Plant-virus-derived VLPs, such as the tobacco mosaic virus (TMV) and cowpea mosaic virus (CPMV), were used as delivery platforms for mRNA vaccine and diagnostic reagent production. A self-amplifying mRNA vaccine packaged by TMV coat proteins expressing E7 protein from human papillomavirus 16 (HPV16) elicits E7-specific IgG antibodies. The same study demonstrated that TMV VLPs are suitable for mRNA vaccine delivery in vivo. Furthermore, plant-derived VLPs are used as nanoparticles to present heterologous immunogens to the immune system, inducing potent cellular and humoral immune responses. These chimeric proteins can self-assemble due to the structural properties of the viral capsid proteins and form particles with similar morphology and structure to the native virion [169,170,171,172]. Table 2 provides the most notable examples of chimeric VLPs produced in plants.

### 4.3. Plant-Derived Antibodies Used for Passive Immunotherapy

Passive immunotherapy is a form of immunization that uses immunoactive products, such as antibodies produced outside the patient’s body. These antibodies provide immediate protection against infections or diseases without necessitating the immune system’s involvement. This approach is particularly beneficial for immunocompromised patients or when a rapid response to a threat is not possible [183]. Antibodies represent a critical component of the vertebrate adaptive immune system. Antibodies can be produced by plants, genetically modified with the genes that encode mammalian and human immunoglobulins. Plant-derived antibodies (plantibodies) offer several advantages, including reduced production costs, the absence of animal pathogens, and scalability. Importantly, plantibodies demonstrate high stability and a long shelf life, making them suitable for application in countries with limited medical infrastructure. Model organisms such as tobacco (*N. benthamiana* and *N. tabacum*), rice, and potato are among the most commonly used plant bioreactors due to their high yield and ease of cultivation. Current approaches focus on increasing expression and shortening production time. The magnification approach, which involves the insertion of multiple provectors into *Agrobacterium tumefaciens*, is a recent development in monoclonal antibody (mAb) production. This method has the potential to yield functional mAbs within 14 days, making it a valuable tool for preclinical, clinical, and commercial applications [184,185,186].

In recent years, plantibodies have gained significant popularity, emerging as an alternative to conventionally used antibodies derived from mammalian cell cultures [187]. Significant parallels have been observed between plant and mammalian antibody production, particularly in terms of glycosylation. In this regard, plants typically add mannose and xylose to N-linked oligosaccharides, whereas animals add galactose and sialic acid. This variation in glycosylation has the potential to impact the immunogenicity and efficacy of plant-derived antibodies when administered to humans [188,189]. Through the use of genetic engineering, modifications have been introduced to enhance glycosylation compatibility with human physiology [190]. Antibodies containing glycans that are nearly homogeneous, possess a mammalian structure, and exhibit enhanced neutralizing capabilities have recently been generated in plants. For example, in the case of antibodies directed against the Ebola virus, the incorporation of a specific motif, such as KDEL, has been shown to direct the antibody to the endoplasmic reticulum, where glycosylation occurs. This results in the formation of N-glycans with a high mannose content, which, in turn, enhances the efficiency of antibody binding to viral particles [191]. In addition, precise control of N-glycosylation was achieved through the generation of transgenic plants (*N. benthamiana*) with reduced xylosyl- and fucosyltransferase (ΔXF) activity by RNA interference. The antibodies expressed in these plants contain homogeneous N-glycans (GnGn), which increases their affinity for FcγRIIIa. This study provides the first successful example of improved antiviral activity by glycoengineering, as evidenced by the production of an antibody against HIV in these plants [192].

The yield and quality of recombinant secretory IgA antibodies can be improved by the engineering of the endoplasmic reticulum (ER) in *N. benthamiana* [193]. Göritzer et al. expanded the ER by targeting the enzyme CTP:phosphocholine cytidylyltransferase (CCT), using CRISPR/Cas to perform site-directed mutagenesis of each of the three endogenous CCT genes in *N. benthamiana*. The mutant plants with a changed ER increased the yields of assembled secretory IgAs due to prolonged ER residence time and a boost in chaperone accumulation [193]. As interest in antibody production from plant systems has grown, so has their productivity. In recent years, several antibodies against some of the more serious viral diseases, such as human immunodeficiency virus, Ebola virus, West Nile virus, dengue virus, herpes simplex virus, rabies virus, and respiratory syncytial virus, have been expressed in plants. Table 3 presents a comprehensive overview of plant antibodies, their expression system, and the infectious diseases targeted.

### 4.4. Recombinant Cytokines Produced in Plants

Cytokines are signaling proteins produced by cells to influence the immune response and help control inflammation in the human body [219]. Recombinant human cytokines have been produced in plants using stable or transient expression methods for increasing protein yield and stability [220]. Interferons, the main group of cytokines, play a central role in the regulation of immune and inflammatory responses to viral infections [221]. Interferon alfa (INF-α) is approved by the FDA in the treatment of chronic hepatitis C and B, and AIDS-related Kaposi’s sarcoma [222]. Recombinant human interferons have been successfully produced in different plant species (carrot, lettuce, potato, and rice) [222,223,224,225,226,227,228,229]. Similarly to the commercial product, a recombinant IFN demonstrated dose-dependent antivirus activity in human A549 cells [223]. These findings show that using scalable and environmentally friendly plant-based expression systems for the production of functional recombinant human proteins is feasible.

### 4.5. Recombinant Carbohydrate-Binding Proteins with Antiviral Activity Produced in Plants

Some native small carbohydrate-binding proteins from plants (lectins) can be used as antivirals. Lectins have the capacity to bind to and neutralize a wide variety of viruses by obstructing the glycan structures found on the surface of the virus [230]. The lectin Griffithsin (GRFT), produced from red algae, has shown broad-range antiviral efficacy against many enveloped viruses, such as HIV-1, SARS-CoV-2, MERS-CoV, HCV, Japanese encephalitis virus (JEV), herpes simplex virus-2 (HSV-2), and porcine epidemic diarrhea virus (PEDV) [231,232]. Lectins can also be obtained via genetically modified plants, thereby affording increased production and simplified purification (Table 4) [233].

## 5. Engineering of Plant Biosynthetic Pathways for Overproduction of Phytochemicals

Numerous therapeutic substances naturally occur in plants. They are based on secondary metabolites (terpenoids, alkaloids, flavonoids, etc.) and can be extracted from their native producers [244]. However, many of them are produced in specific plant tissues or at certain developmental stages. This specific spatiotemporal expression pattern limits the overall yield when attempting to extract metabolites from the entire plant [9]. Plant secondary metabolites are produced via multistep biosynthetic pathways that involve a variety of enzymes, cofactors, and intermediates that have a highly specific stereochemistry. In recent years, research has increasingly focused on extensively characterizing the biosynthetic pathways of plant-derived secondary metabolites, driven by the need to produce these metabolites on a larger scale and in a more controlled and economically viable manner.

### 5.1. Improved Production of Phytochemicals by CRISPR-Cas9 Genome Editing

In recent years, genome editing technologies have significantly advanced our ability to manipulate plant metabolic pathways for the production of valuable bioactive compounds. CRISPR-Cas9 technology enables the targeted editing of specific genes involved in plant biosynthetic pathways. Key strategies include gene knockout, multiplex editing, and targeted mutations to optimize the biosynthetic pathways of important compounds such as alkaloids and phenols. For instance, *Salvia miltiorrhiza* has been genetically edited to enhance its phenolic acid content [245]. The bZIP2 gene was knocked out using CRISPR-Cas9, leading to an increased concentration of phenolic acids. In another case, *Cichorium intybus* (chicory), which produces sesquiterpene lactones (STLs), a group of compounds known for their antiviral and anticancer properties [246], was edited using CRISPR-Cas9 to inhibit the germacrene A synthase (CiGAS) enzyme. This led to an upregulation of phenolic acid production, enhancing the plant’s antiviral potential [247]. The applications of CRISPR/Cas9 in medicinal plants to improve the production of bioactive compounds are summarized in Table 5.

Prior to the advent of CRISPR/Cas technology, various non-CRISPR/Cas genetic manipulation techniques were employed to enhance the production of specialized metabolites in medicinal plants. These methods include gene silencing with RNA interference (RNAi), gene stacking (multiple genetic modifications in a single plant), and overexpression [255].

### 5.2. Transient Expression of Biosynthetic Enzymes

The transient expression of biosynthetic enzymes in *N. benthamiana* has emerged as a powerful approach for the production of complex plant-derived metabolites. This method has become a “go-to” strategy due to its speed and flexibility, allowing researchers to bypass the slower and more labor-intensive process of stable transformation [256,257]. Such successful transient expression is exemplified by the production of triterpenes, e.g., saponins, in *N. benthamiana*. A combinatorial approach was used to co-express 16 enzymes involved in the biosynthesis of triterpenes. This system enabled the production of a saponin molecule that could potentially be further engineered as an adjuvant for vaccines [258].

## 6. Challenges and Limitations of Plant-Derived Antivirals and Recombinant Proteins

Plant-derived natural products and advancements in plant biotechnology provide effective approaches in the global fight against emerging viral threats [259]. Unlike synthetic drugs, which are usually designed with a specific target in mind, products extracted from plants often exhibit broad-spectrum antiviral activity. Their inherent multitarget capability allows them to inhibit several viral proteins or pathways simultaneously, reducing the likelihood of drug resistance. However, this benefit comes with the increased risk of off-target effects, necessitating deeper investigation into the mechanisms of action, therapeutic effects, and potential toxicity of these products. Detailed preclinical therapeutic and toxicological profiling of potential therapeutic agents of natural origin is essential to mitigate these risks and support the safe advancement of these compounds into clinical use.

In terms of production, sustainable sourcing remains a major challenge. Medicinal plants often require specific conditions and extended periods for cultivation, making scaling difficult, especially in times of urgency, such as pandemics. However, recent breakthroughs in plant genomics and metabolomics have supported the development of biotechnological approaches to produce these valuable natural compounds in engineered plant systems [260,261,262].

Plant molecular farming has already demonstrated success in addressing the COVID-19 pandemic, using plants to produce the SARS-CoV-2 virus-like particle vaccine, SARS-CoV-2 neutralizing antibodies, and diagnostic reagents [207,263,264]. Conventional plant transformation methods are constrained by genotype-specific limitations and long development cycles. As a result, there is growing interest in transient expression systems in plants, which offer faster and more flexible alternatives for producing bioactive compounds and proteins. The achievements of PMF highlight the viability of plants as pharmaceutical protein production platforms, boosting confidence in their broader application [265,266]. However, the purification and downstream processing of plant-derived proteins remain significant bottlenecks due to their complexity and high cost. One of the most promising future directions is the development of oral vaccines that bypass the need for protein purification altogether [267]. Moreover, as peroral vaccines face the hurdle of protein degradation in the digestive tract, inhalable formulations offer the advantage of localized immune activation against viruses [268,269,270].

## 7. Conclusions

Plant-derived natural products and biotechnological innovations offer a multifaceted strategy to combat emerging viral threats. By addressing challenges in specificity, supply chain, biosynthesis, and delivery, plant-based solutions could play a transformative role in future pandemic preparedness and response. Continued interdisciplinary research is critical to unlock their full potential and ensure safety, efficacy, scalability, and sustainability. As explored in this review, an integrated approach combining genome editing, structural biology, metabolic engineering, and gene engineering can significantly enhance the development and utilization of plant-based antiviral agents. Despite the inherent challenges, such as production constraints and the complexity of biosynthetic pathways, continued innovation in these areas holds great potential for generating effective plant-derived drugs, vaccines, antibodies, and diagnostic tools. The lessons learned from the COVID-19 pandemic and technologies developed during that period underscore the importance of adaptable technologies for vaccine and antiviral production. The progress made not only provides a foundation for addressing current health crises but also serves as a strategic framework for mitigating future viral outbreaks.

## Figures and Tables

**Table 1:** Examples of plant-derived vaccines to prevent recently emerging viruses.

Virus	Antigen	Production System	Immunogenicity	Company
SARS-CoV-2	Modified VLPs built from S protein	*N. benthamiana*/transient	Phase-3 clinical trial completed: 71% efficacy rate of VLP plant-based vaccine against all variants of SARS-CoV-2	Medicago Inc. [162]
	KBP-201 CoVLPs targeting RBD	*N. benthamiana*/transient	Phase-1/2 clinical trials	Kentucky Bioprocessing (KBP) [150]
	Subunit Vax 1	*N. benthamiana*/transient	Phase-1/2 clinical trials	Baiya Phytopharm [149]
Zika virus	Zika virus envelope protein (zE)-based candidate subunit vaccine	*N. benthamiana*/transient	Elicited potent zE-specific antibodies and cellular immune responses in mice	Academic research [163]
Chikungunya virus	VLPs (full-length envelope protein E2)	*N. benthamiana/transient*	Preclinical, demonstrating feasibility of CHIKV E2 expression in plants	Academic research [157]
Ebola virus	Multiepitopic protein Zerola (GP epitopes)	*N. tabacum*/stable gene expression	No data	Academic research
	Ebola glycoprotein (GP) in fusion with 6D8 anti-Ebola IgG (6D8 IgG-GP1)	*N. benthamiana*/transient	80% survival of immunized mice (SC) against lethal EBV challenge [164]	
	EBOV GP1 in fusion with *E. coli* heat-labile enterotoxin B subunit (LTB-EBOV)	Tobacco/transgenic (nuclear)	IgA and IgG responses induced following oral immunization [165]	
Influenza	VLPs composed only of influenza hemagglutinin (HA)-H5	*N. benthamiana*/transient	Elicited potent humoral and cell-mediated immune responses to H5N1	Medicago Inc. [166]
	Polyvalent HA VLPs for seasonal flu	*N. benthamiana*/transient	Successful completion of phase-3 clinical studies for plant-derived VLP quadrivalent flu vaccine	Medicago Inc. [167]

**Table 2:** Chimeric VLPs displaying foreign antigens and their immunogenic properties.

Virus Used for VLP Production	Foreign Antigen Displayed on VLPs	Plant/Expression Method	Expression per Fresh Weight	Immune Responses/References
CPMV	VP2 capsid protein from mink enteritis virus (MEV)	*Vigna unguiculata*/ virus transfection	1 mg/g	A total of 1 mg of the CVPs in mink protected against clinical illness and prevented virus shedding following exposure to virulent MEV [173].
TMV	L2 epitopes from cottontail rabbit papillomavirus and rabbit oral papillomavirus	*N. benthamiana*/ transient	No data	Rabbits immunized with the chimeric VLPs were protected from developing papillomas [174].
Alfalfa mosaic virus coat protein (AMV)	Pfs25 protein of *Plasmodium falciparum*	*N. benthamiana*/ transient	50 µg/g	In a phase-I study, IgG responses > 3 log10 were observed with a dose of 100 μg [175].
Bamboo mosaic virus-based (BaMV)	Foot-and-mouth disease virus (FMDV) VP1 antigens	*N. benthamiana*/ transient	No data	No immunization studies have been reported.
Bacteriophage AP 205	Envelope Protein Domain III (EDIII) of WNV	*N. benthamiana*/ transient	36 µg/g purified yield	A total of 5 μg of chimeric VLPs elicited IgG responses in immunized mice [175].
Hepatitis B virus (HBV)	Chimeric VLPs based on HBcAg that presented zDIII	*N. benthamiana*/ transient	1824 μg/g	They elicit a potent humoral and cellular immune response in mice [176].
	Chimeric VLPs presenting M2e of influenza		1–2% of total soluble protein	Chimeric VLPs have a protective effect against a lethal influenza challenge in mice [177].
	Chimeric VLPs bearing epitope of HEV capsid		VLP recovery yield of 10 µg/g	No immunization studies have been reported [178].
Hepatitis E virus (HEV)	M2e of influenza	*N. benthamiana*/ transient	300 µg/g	They do not induce a protective immune response in mice [171,179,180].
	RBD of SARS-CoV-2		100 µg/g	HEV/RBD chimeric proteins are recognized in human serum from COVID-19 patients [170].
Bluetongue virus (BTV)	EDIII of dengue virus and Zika virus	*N. benthamiana*/ transient	5–15 µg/g purified yield	They induce a humoral immune response in mice [181].
African horse sickness virus (AHSV)	VP2, VP3, VP5, and VP7 from AHS serotype 1; VP2 and VP5, serotype 7; VP5, serotype 3, and VP2, serotype 6	*N. benthamiana*/ transient	No data	They elicit a weak neutralizing humoral immune response in these target animals against homologous AHSV virus [182].

**Table 3:** Overview of plant antibodies, the system in which they are expressed, and the infectious diseases against which they are directed.

Disease/Pathogen	Antibody	Plant System	Reference
HIV	2G12; 2F5; b12: b12-CV-N; 10-1074, VRC01; 3BNC117; 4E10	*N. benthamiana*, *N. tabacum*, *Z. mays* (maize), *A. thaliana*, *O. sativa* L. (rice)	[194,195,196,197,198,199,200]
Ebola	h-13F6, ZMapp™, anti-Ebola monoclonal antibodies	*N. benthamiana*, *N. tabacum*	[191,200,201]
West Nile Virus	Hu-E16 mAb, HPA	*N. benthamiana*	[202,203]
Hepatitis B virus	Anti-HB Ab	*N. tabacum*, *S. lycopersicum* L. (tomato), *S. tuberosum* L. (potato), *L. sativa* L. (lettuce), *Banana. cv. Rasthali*	[204,205]
SARS-CoV-2	Anti-SARS-CoV-2 Ab, mAbJ08-MUT, mAb675, B38, H4	*N. benthamiana*	[206,207,208]
Rabies	Anti-rabies Ab	*N. benthamiana*	[209]
Respiratory syncytial virus	Anti-RSV Ab	*N. benthamiana*	[210]
Rotavirus	Anti-Rotavirus Ab	*N. benthamiana*, *S. lycopersicum* L., *S. tuberosum* L., *M. sativa* L. (Alfalfa), *O. sativa* L.	[211,212,213,214]
Dengue virus	Anti-dengue Ab	*N. benthamiana*	[215,216]
CHIKV	Five anti-chikungunya neutralizing monoclonal antibodies (mAbs)	*N. benthamiana*	[217]
Zika virus	Anti-Zika mAb	*N. benthamiana*	[218]

**Table 4:** Recombinant lectins produced in plants.

Plant System/Expression Method	Lectin Yield	Virus Neutralization Activity/Reference
*N. tabacum*, stable chloroplast transformation	GRFT up to 5% of TSP of plant; yield of 360 µg/g of fresh weight (FW).	It demonstrated similar anti-HIV activity to GRFT expressed in bacteria [234].
*N. benthamiana*, transient expression	Final recovery of 30% of in planta level of 1 g/kg.	GRFT-P showed broad-spectrum activity against HIV [235].
Transgenic rice (*O. sativa*), endosperm	GRFT up to 223 μg/g dry seed W, recovery of 74%.	It binds to HIV glycans with similar efficiency to GRFT produced in *E. Coli* [236].
*N. tabacum*, stable transformation	Cyanovirin-N (CV-N) recoverable at levels of 130 ng/mg of FW.	CV-N bound to soluble gp120IIIb in a concentration-dependent manner [237].
Transgenic roots of marshmallow plant (*Althaea officinalis* L.)	Concentration of CV-N in root tissue of 2.4 lg/g FW.	An ELISA plate coated with gp120 confirmed the functionality of the CV-N [238].
Transgenic soya bean plants	CV-N with yield of 350 μg/g of dry seed weight, 92% purity.	Purified rCV-N is active in anti-HIV assays with an EC_50_ of 0.82–2.7 nM [239].
Transgenic rice plants (*O. sativa*), endosperm	Yield up to 10 µg CV-N per gram dry seed.	The crude extracts showed dose-dependent gp120-binding activity [240].
Transgenic *N. tabacum* plants	Expression of fusion protein consisting of mAb b12/CV-N.	Each moiety of the fusion protein retained its binding ability to gp120 [241].
Transgenic rice plants (*O. sativa*), endosperm	2G12, GRFT, and CV-N expressed simultaneously with varying yields.	Extracts of transgenic plants expressing all three proteins showed enhanced in vitro binding to gp120 [242].
*N. benthamiana*, transient expression	Fusion VRC01_Fab_–Avaren protein with yield of 40 mg/kg of FW.	NVRC01_Fab_–Avaren showed stronger HIV-1 neutralization activity [243].

**Table 5:** Modification of medicinal plants to improve the production of bioactive compounds using CRISPR/Cas9 technology for gene editing.

**Plant Species**	**Metabolite(s)**	**Potential Antiviral Activity**	**Target Gene Edited/References**
*Salvia miltiorhiza* (red sage/Danshen)	Tanshinones	Tanshinone I acts as a cap-dependent endonuclease inhibitor.	CPS1 (tanshinone biosynthesis pathway) [248].
*Cannabis sativa* (hemp)	THC, THC-free related metabolites	Cannabinoids show potential antiviral activity against HIV and herpes simplex.	CsPDS (to generate THC-free phenotype) [249,250].
*Dendrobium officinale* (Dendrobium orchid)	Polysaccharides, bibenzyls	Polysaccharides from *D. officinale* have immune-boosting effects.	C3H, C4H, 4CL, CCR, IRX (metabolic pathway genes) [251,252].
*Artemisia annua* (sweet wormwood)	Artemisinin	Artemisinin has potential antiviral properties against HIV and HBV.	SQS (Squalene synthase, competitive with artemisinin) [253,254].

## Data Availability

Not applicable.

## Abbreviations

The following abbreviations are used in this manuscript:

SARS-CoV	Severe Acute Respiratory Syndrome Coronavirus
SARS-CoV-2	Severe Acute Respiratory Syndrome Coronavirus 2
MERS-CoV	Middle East Respiratory Syndrome Coronavirus
CoVs	Coronaviruses
H1N1	Hemagglutinin Type 1 and Neuraminidase Type 1 (Influenza A subtype)
HIV-1	Human Immunodeficiency Virus Type 1
mRNA	Messenger Ribonucleic Acid
VLPs	Virus-Like Particles
IVA	Influenza Virus A
DENV	Dengue Virus
ZIKV	Zika Virus
EBOV	Ebola Virus
MPXV	Monkeypox Virus
RNA	Ribonucleic Acid
RNP	Ribonucleoprotein
DNA	Deoxyribonucleic Acid
S protein	Spike Protein
ACE2	Angiotensin-Converting Enzyme 2
DPP4	Dipeptidyl Peptidase 4
TMPRSS2	Transmembrane Protease Serine 2
NP	Nucleoprotein
VP	Viral Protein
GP	Glycoprotein
sGP	Soluble GP
L	Polymerase
NTPase	Nucleoside Triphosphatase
HA	Hemagglutinin
vRNP	Viral Ribonucleoprotein Complex
E protein	Envelope Protein
CD4	Cluster of Differentiation 4
CCR5	C-C Chemokine Receptor Type 5
CXCR4	C-X-C Chemokine Receptor Type 4
NPC1	Niemann–Pick C1 Protein
E8L	A Viral Envelope Protein from MPXV
EGCG	Epigallocatechin Gallate
ASA, ASAI	Allium Sativum Lectins
IC_50_	Half Maximal Inhibitory Concentration
FRET	Förster Resonance Energy Transfer
Mpro/3CLpro	Main Protease/3-Chymotrypsin-Like Protease (also nsp5)
PLpro	Papain-Like Protease (also nsp3)
NSPs	Non-Structural Proteins
RdRp	RNA-Dependent RNA Polymerase (also nsp12)
RTC	Replication–Transcription Complex
ER	Endoplasmic Reticulum
ERGIC	Endoplasmic Reticulum–Golgi Intermediate Compartment
IRES	Internal Ribosome Entry Site
RT	Reverse Transcriptase
GAGs	Glycosaminoglycans
DC-SIGN/L-SIGN	Dendritic Cell-Specific Intercellular Adhesion Molecule-3-Grabbing Non-Integrin/Liver/Lymph Node-Specific ICAM-3-Grabbing Integrin
TIM-1	T-cell Immunoglobulin and Mucin-Domain-Containing-1
PI3K/Akt	Phosphoinositide 3-Kinase/Protein Kinase B pathway
ELISA	Enzyme-Linked Immunosorbent Assay
PMF	Plant Molecular Farming
DSP	Downstream Processing
HIV	Human Immunodeficiency Virus
TMV	Tobacco Mosaic Virus
CPMV	Cowpea Mosaic Virus
HPV16	Human Papillomavirus 16
AMV	Alfalfa Mosaic Virus
BaMV	Bamboo Mosaic Virus-Based
FMDV	Foot-and-Mouth Disease Virus
AP 205	Bacteriophage AP 205
HBV	Hepatitis B Virus
HEV	Hepatitis E Virus
BTV	Bluetongue Virus
AHSV	African Horse Sickness Virus
mAb	Monoclonal Antibody
KDEL	Retention Signal Sequence in Proteins
ΔXF	Deletion of Xylosyl- and Fucosyltransferase (enzyme activity)
CTP	Cytidine Triphosphate
CCT	CTP:phosphocholine cytidylyltransferase
CRISPR/Cas	Clustered Regularly Interspaced Short Palindromic Repeats/CRISPR-Associated Protein
IgA	Immunoglobulin A
INF-α	Interferon Alfa
FDA	Food and Drug Administration
GRFT	Griffithsin
CV-N	Cyanovirin-N
EC_50_	Half Maximal Effective Concentration
STLs	Sesquiterpene Lactones
CiGAS	Germacrene A Synthase from Cichorium

## Appendix A

**Table A1:** Plant-derived compounds affecting viral adsorption, fusion, and entry.

Compound	Activity	Cell Type Tested	Target	IC_50_/EC_50_	Plant	Reference
**SARS-CoV-2**						
Cepharantine	Inhibition of pre-entry, entry, and membrane fusion	Calu-3, A549, HEK293T-ACE2, Vero E6	Blockage of host calcium channels	0.315 µM	N/A	[23]
Hernandezine	Inhibition of pre-entry, entry, and membrane fusion	Calu-3, A549, HEK293T-ACE2, Vero E6	Blockage of host calcium channels	0.111 µM	N/A	[23]
Neferine	Inhibition of pre-entry, entry, and membrane fusion	Calu-3, A549, HEK293T-ACE2, Vero E6	Blockage of host calcium channels	0.946 µM	N/A	[23]
ASA	Inhibition of early viral attachment	Vero E6	N/A	>4 µM	*Allium sativum* L.	[271]
ASA1	Inhibition of early viral attachment	Vero E6	N/A	>4 µM	*Allium sativum* L.	[271]
Punicalin	Inhibition of viral entry	N/A	Disruption of spike glycoprotein–host ACE2 interaction	0.009 µM	*Gunnera perpensa*	[25]
Punicalagin	Inhibition of viral entry	N/A	Disruption of spike glycoprotein–host ACE2 interaction	0.029 µM	*Gunnera perpensa*	[25]
Epigallocatechin gallate	Inhibition of viral entry	HEK293FT, Caco-2	Disruption of spike glycoprotein–host ACE2 interaction	33.9 µM	*Camelia sinensis*	[28]
4,6-dihydroxyquinoline-2-carboxylic acid	Inhibition of viral entry	Calu-3, HEK293T-ACE2	Disruption of spike glycoprotein–host ACE2 interaction	0.07 µM	*Ephedra sinica*	[26]
4-hydroxy-6-methoxyquinoline-2-carboxylic acid	Inhibition of viral entry	Calu-3, HEK293T-ACE2	Disruption of spike glycoprotein–host ACE2 interaction	0.15 µM	*Ephedra sinica*	[26]
4-hydroxyquinoline-2-carboxylic acid	Inhibition of viral entry	Calu-3, HEK293T-ACE2	Disruption of spike glycoprotein–host ACE2 interaction	0.58 µM	*Ephedra sinica*	[26]
**Dengue virus**						
Gossypol	Inhibition of viral attachment	LLC-MK2	Envelope protein region III	1.87 µM (DENV-1) 1.89 µM (DENV-2) 3.7 µM/(DENV-3) 2.6 µM/ (DENV-4)	*Gossypium* spp.	[38]
Baicalein	Inhibition of DENV-2 adsorption	Vero E6	Not established	7.14 μg/mL	*Scutellaria baicalensis*	[35]
Baicalin	Inhibition of viral adsorption	Vero E6	Not established	18.07 μg/mL	*Scutellaria baicalensis*	[37]
**Zika virus**						
Baicalin	Inhibition of viral attachment to host cells	Vero E6	Envelope E protein	14 µM	*Scutellaria baicalensis*	[36]
Gossypol	Inhibition of viral attachment to host cells	Vero E6	Envelope protein region III	22.2 µM	*Gossypium* sp.	[38]
Curcumin	Inhibition of viral attachment to host cells	HeLa, BHK-21, Vero E6	Envelope E protein	1.90 µM	*Curcuma longa*	[40]
(−) Epigallocatechin gallate	Destabilization and dissolution of viral particle	Vero E6	Viral envelope phospholipids	21.4 µM	*Camellia sinensis*	[39]
Isoquercitrin	Inhibition of membrane-associated viral particle internalization into A549 cells	A549	Not established	15.5 µM	*Mangifera indica*	[41]
**HIV-1**						
Ajoene	Inhibition of adhesive interactions and fusion of leukocytes	T-lymphoblasts (H9, CEM13)	N/A	45 µM	*Allium* *sativum* L.	[272]
“TFmix” (theaflavin; theaflavin-3-gallate; theaflavin-3′-gallate; theaflavin-3-3′-digallate)	Inhibition of viral attachment	H9/HIV-1_IIIB_ cells, MT-2	Interference with viral gp41 6-helix bundle formation	6.25 µM	*Camellia sinensis* *assamica*	[273]
Cassiabrevone	Inhibition of viral attachment	U373-CD4-CXCR4	Viral gp120-host CD4 binding	30.96 µM	*Cassia abbreviata*	[274]
Acerosin	Inhibition of viral entry	MT-2	Surmised blocking of virus–CD4 or CXCR4/CCR5 host cell receptor interaction	2.7 µM	*Artemisia campestris*	[275]
Xanthomicrol	Inhibition of viral entry	MT-2	Surmised blocking of virus–CD4 or CXCR4/CCR5 host cell receptor interaction	17.43 µM	*Artemisia campestris*	[275]
Guibourtinidol-(4α → 8)-epiafzelechin	Inhibition of viral attachment	U373-CD4-CXCR4	Viral gp120-host CD4 binding	42.47 µM	*Cassia abbreviata*	[274]
Baicalin	Inhibition of viral fusion	Hos/CD4/CCR5, Hos/CD4/CXCR4	Broad inhibition of T-cell tropic (X4) and monocyte tropic (R5) HIV-1 Env protein-mediated fusion with host CD4/CXCR4 or CD4/CCR5	4 µM	*Scutellaria baicalensis*	[32]
Procyanidin A (pentamer)	Inhibition of viral attachment	PBMC	Blocks binding of viral gp120 to host heparan sulfate	7 µM	*Cinnamomum cassia*	[276]
Procyanidin A (trimer)	Inhibition of viral attachment	PBMC	Blocks binding of viral gp120 to host heparan sulfate	7.5 µM	*Cinnamomum cassia*	[276]
Procyanidin A (pentamer)	Inhibition of viral attachment	PBMC	Blocks binding of viral gp120 to CD4	21.5 µM (YU2 HIV-1 envelope), 20 µM (MN HIV-1 envelope)	*Cinnamomum cassia*	[276]
**Ebola virus**						
(+) Catechin	Inhibition of viral attachment, entry, and fusion	A549, HEK293T	Blocking Ebola glycoprotein	36.0 µM	*Maesa perlarius*	[59]
Ellagic acid	Inhibition of viral entry	A549, HeLa	Blocking Ebola glycoprotein-mediated entry	1.4 µM (against pseudovirions in A549 cells) 10.5 µM (against EBOV in HeLa cells)	*Rhodiola rosea* L.	[277]
(−) Epicatechin	Inhibition of viral attachment, entry, and fusion	A549, HEK293T	Blocking Ebola glycoprotein	22.1 µM	*Maesa perlarius*	[59]
Epicatechin gallate	Inhibition of viral attachment, entry, and fusion	A549, HEK293T	Blocking Ebola glycoprotein	2.95 µM	*Maesa perlarius*	[59]
Epigallocatechin	Inhibition of viral attachment, entry, and fusion	A549, HEK293T	Blocking Ebola glycoprotein	5.53 µM	*Maesa perlarius*	[59]
Epigallocatechin-3-gallate	Inhibition of viral attachment, entry, and fusion	A549, HEK293T	Blocking Ebola glycoprotein	2.8 µM	*Maesa perlarius*	[59]
Gallocatechin	Inhibition of viral attachment, entry, and fusion	A549, HEK293T	Blocking Ebola glycoprotein	12.4 µM	*Maesa perlarius*	[59]
Procyanidin B1	Inhibition of viral attachment, entry, and fusion	A549, HEK293T	Blocking Ebola glycoprotein	0.95 µM	*Maesa perlarius*	[59]
Procyanidin B2	Inhibition of viral attachment, entry, and fusion	A549, HEK293T	Blocking Ebola glycoprotein	0.83 µM	*Maesa perlarius*	[59]
Quercetin 3-β-O-d-glucoside	Inhibition of early stages of viral entry	Vero E6	Not established, surmised involvement of NPC1 endosomal transporter/LDL receptors	5.3 µM	N/A	[60]
**Influenza A**						
Harmalol	Viricidal and antivirus adsorption effects	MDCK	Not established	0.035 µg/mL	*Peganum harmala* L.	[56]
Harmane	Viricidal and antivirus adsorption effects	MDCK	Not established	0.033 µg/mL	*Peganum harmala* L.	[56]
Harmaline	Viricidal and antivirus adsorption effects	MDCK	Not established	0.056 µg/mL	*Peganum harmala* L.	[56]
Strychnine sulfate	Viricidal and antivirus adsorption effects	MDCK	Not established	0.06 µg/mL	*Peganum harmala* L.	[56]
Pentagalloylglucose	Inhibition of viral adsorption	MDCK	Viral hemagglutinin	2.51 µM	*Phyllanthus emblica Linn*	[278]
5,7,3′,4′-tetra-O-methylquercetin	Blocking host cell entry and/or recognition	MDCK	Binding to H1N1 virions	0.36 µM	*Sambucus nigra* L.	[54]
(±)-dihydromyricetin	Blocking host cell entry and/or recognition	MDCK	Binding to H1N1 virions	8.7 µM	*Sambucus nigra* L.	[54]
Cyanidin 3-sambubioside	Inhibition of viral adsorption	MDCK	Not established	252 µg/mL (whole-extract value)	*Sambucus nigra* L.	[54]
Quercetin	Inhibition of viral entry	MDCK	HA2 subunit of influenza hemagglutinin	7.76 µM (*A/Puerto Rico/8/34* (*H1N1*)), 6.23 µM (*A/FM-1/47/1* (*H1N1*)), 2.74 µM (*A/Aichi/2/68* (*H3N2*))	N/A	[53]
**Chikungunya virus**						
Epigallocatechin gallate	Inhibition of viral entry	HEK293T	Envelope glycoprotein	12 µM	*Camellia Sinensis*	[47]
Baicalein	Inhibition of viral adsorption	Vero cells	Not established	103.76 µM	*Scutellaria baicalensis*	[48]
Quercetagetin	Inhibition of viral adsorption	Vero cells	Not established	25.3 µM	N/A	[48]
Curcumin	Affects viral glycoprotein conformation and/or membrane fluidity	HeLa	Viral envelope	3.89 µM	*Curcuma longa*	[40]
2-(butoxycarbonyl) benzoic acid (BCB)	Inhibition of viral entry	Vero cells	E1 CHIKV envelope glycoprotein	2.49 µg/mL vs. Asian isolate 28.62 µg/mL vs. African isolate	*Tectona grandis*	[50]
3,7,11,15-tetramethyl-1-hexadecanol (THD)	Inhibition of viral entry	Vero cells	E1 CHIKV envelope glycoprotein	1.66 µg/mL vs. Asian isolate 122.4 µg/mL vs. African isolate	*Tectona grandis*	[50]
Benzene 1-carboxylic acid hexadecanoate (BHCD)	Inhibition of viral entry	Vero cells	E1 CHIKV envelope glycoprotein	3.04 µg/mL vs. Asian isolate 76.46 µg/mL vs. African isolate	*Tectona grandis*	[50]

**Table A2:** Plant-derived compounds targeting specific proteins involved in viral replication and maturation.

Compound	Target	Cell Type Tested	IC_50_/EC_50_	Plant	Reference
**Coronaviruses** **(SARS-CoV, SARS-CoV-2, MERS-CoV)** **3CLPro**					
3′-(3-Methylbut-2-enyl)-3′,4′,7-trihydroxyflavane	MERS-CoV-3CLPro	N/A	34.7 µM	*Broussonetia papyrifera*	[72]
4-Hydroxyisolonchocarpin	MERS-CoV-3CLPro	N/A	193.7 µM	*Broussonetia papyrifera*	[72]
Broussochalcone A	MERS-CoV-3CLPro	N/A	36.2 µM	*Broussonetia papyrifera*	[72]
Broussochalcone B	MERS-CoV-3CLPro	N/A	27.9 µM	*Broussonetia papyrifera*	[72]
Broussoflavan A	MERS-CoV-3CLPro	N/A	125.7 µM	*Broussonetia papyrifera*	[72]
Isoliquiritigenin	MERS-CoV-3CLPro	N/A	33.9 µM	*Broussonetia papyrifera*	[72]
Kaempferol	MERS-CoV-3CLPro	N/A	35.3 µM	*Broussonetia papyrifera*	[72]
Kazinol A	MERS-CoV-3CLPro	N/A	66.2 µM	*Broussonetia papyrifera*	[72]
Kazinol B	MERS-CoV-3CLPro	N/A	31.4 µM	*Broussonetia papyrifera*	[72]
Kazinol F	MERS-CoV-3CLPro	N/A	135.0 µM	*Broussonetia papyrifera*	[72]
Kazinol J	MERS-CoV-3CLPro	N/A	109.2 µM	*Broussonetia papyrifera*	[72]
Papyriflavonol A	MERS-CoV-3CLPro	N/A	64.5 µM	*Broussonetia papyrifera*	[72]
Quercetin	MERS-CoV-3CLPro	N/A	34.8 µM	*Broussonetia papyrifera*	[72]
Quercetin-β-galactoside	MERS-CoV-3CLPro	N/A	68.0 µM	*Broussonetia papyrifera*	[72]
3′-(3-Methylbut-2-enyl)-3′,4′,7-trihydroxyflavane	SARS-CoV-3CLPro	N/A	30.2 µM	*Broussonetia papyrifera*	[72]
3-isotheaflavin-3-gallate	SARS-CoV-3CLPro	N/A	7 µM	*Camellia sinensis*	[80]
4-Hydroxyisolonchocarpin	SARS-CoV-3CLPro	N/A	202.7 µM	*Broussonetia papyrifera*	[72]
Aloe emodin	SARS-CoV-3CLPro	Vero cells	132 μM	*Isatis Indigotica*	[279]
Amentoflavone	SARS-CoV-3CLPro	N/A	8.3 μM	*Torreya nucifera*	[73]
Apigenin	SARS-CoV-3CLPro	N/A	280.8 μM	*Torreya nucifera*	[73]
beta-sitosterol	SARS-CoV-3CLPro	Vero cells	115 μM	*Isatis indigotica*	[279]
Betulinic acid	SARS-CoV-3CLPro	Vero E6	8.2 μM	*Betula pubescens*	[280]
Broussochalcone A	SARS-CoV-3CLPro	N/A	88.1 µM	*Broussonetia papyrifera*	[72]
Broussochalcone B	SARS-CoV-3CLPro	N/A	57.8 µM	*Broussonetia papyrifera*	[72]
Broussoflavan A	SARS-CoV-3CLPro	N/A	92.4 µM	*Broussonetia papyrifera*	[72]
Chalcone	SARS-CoV-3CLPro	N/A	11.4 µM	*Angelica keiskei*	[72]
Daidzein	SARS-CoV-3CLPro	Vero cells	105 μM	*Isatis Indigotica*	[279]
Dihydrotanshinone I	SARS-CoV-3CLPro	N/A	14.4 µM	*Salvia miltiorrhiza*	[281]
Herbacetin	SARS-CoV-3CLPro	N/A	33.17 µM	*Rhodiola Rosea*	[282]
Hesperetin	SARS-CoV-3CLPro	Vero cells	60 μM	*Isatis Indigotica*	[279]
Hirsutanolol	SARS-CoV-3CLPro	N/A	105.6 µM	*Alnus japonica*	[283]
Hirsutenone	SARS-CoV-3CLPro	N/A	36.2 µM	*Alnus japonica*	[283]
Indigo	SARS-CoV-3CLPro	Vero cells	300 μM	*Isatis indigotica*	[279]
Indirubin	SARS-CoV-3CLPro	Vero cells	293 μM	*Isatis indigotica*	[279]
Isoliquiritigenin	SARS-CoV-3CLPro	N/A	61.9 µM	*Broussonetia papyrifera*	[72]
Kaempferol	SARS-CoV-3CLPro	N/A	116.3 µM	*Broussonetia papyrifera*	[72]
Kazinol A	SARS-CoV-3CLPro	N/A	84.8 µM	*Broussonetia papyrifera*	[72]
Kazinol B	SARS-CoV-3CLPro	N/A	233.3 µM	*Broussonetia papyrifera*	[72]
Kazinol F	SARS-CoV-3CLPro	N/A	43.3 µM	*Broussonetia papyrifera*	[72]
Kazinol J	SARS-CoV-3CLPro	N/A	64.2 µM	*Broussonetia papyrifera*	[72]
Methyl tanshinoate	SARS-CoV-3CLPro	N/A	21.1 µM	*Salvia miltiorrhiza*	[281]
Oregonin	SARS-CoV-3CLPro	N/A	129.5 µM	*Alnus japonica*	[281]
Papyriflavonol A	SARS-CoV-3CLPro	N/A	103.6 µM	*Broussonetia papyrifera*	[72]
Pectolinarin	SARS-CoV-3CLPro	N/A	37.78 µM	*Cirsium heterophyllum*	[282]
Quercetin	SARS-CoV-3CLPro	N/A	23.8 μM	*Torreya nucifera*	[73]
Quercetin-β-galactoside	SARS-CoV-3CLPro	N/A	128.8 µM	*Broussonetia papyrifera*	[72]
Rhoifolin	SARS-CoV-3CLPro	N/A	27.45 µM	*Citrus limon*	[282]
Rosmariquinone	SARS-CoV-3CLPro	N/A	21.1 µM	*Salvia miltiorrhiza*	[281]
Rubranol	SARS-CoV-3CLPro	N/A	144.6 µM	*Alnus japonica*	[283]
Rubranoside A	SARS-CoV-3CLPro	N/A	102.1 µM	*Alnus japonica*	[283]
Rubranoside B	SARS-CoV-3CLPro	N/A	105.3 µM	*Alnus japonica*	[283]
Sinigrin	SARS-CoV-3CLPro	Vero cells	121 μM	*Isatis indigotica*	[279]
Tannic acid	SARS-CoV-3CLPro	N/A	3 µM	*Rhus Coriaria*	[80]
Tanshinone I	SARS-CoV-3CLPro	N/A	38.7 µM	*Salvia miltiorrhiza*	[281]
Tanshinone IIA	SARS-CoV-3CLPro	N/A	89.1 µM	*Salvia miltiorrhiza*	[281]
Tanshinone IIB	SARS-CoV-3CLPro	N/A	24.8 µM	*Salvia miltiorrhiza*	[281]
Theaflavin	SARS-CoV-3CLPro	N/A	56.0 µM	*Camellia Sinensis*	[80]
Theaflavin-3,3′-digallate	SARS-CoV-3CLPro	N/A	9.5 µM	*Camellia Sinensis*	[80]
Baicalein	SARS-CoV-2-3CLPro	N/A	0.94 μM	*Scutellaria baicalensis*	[67]
Baicalin	SARS-CoV-2-3CLPro	N/A	6.41 μM	*Scutellaria baicalensis*	[67]
β-carotene	SARS-CoV-2-3CLPro	N/A	17.54 μM	*Vaccinium Oxycoccos*	[82]
Cyanidin 3-O-galactoside	SARS-CoV-2-3CLPro	N/A	9.98 μM	*Vaccinium Oxycoccos*	[82]
Epicatechin	SARS-CoV-2-3CLPro	N/A	12.54 μM	*Vaccinium Oxycoccos*	[82]
Isoschaftoside	SARS-CoV-2-3CLPro	N/A	30.22 μM	*Camellia Sinensis*	[74]
Kaempferol-3-O-gentiobioside	SARS-CoV-2-3CLPro	N/A	35.89 μM	*Camellia Sinensis*	[74]
Narcissoside	SARS-CoV-2-3CLPro	N/A	38.14 μM	*Zygophyllum simplex*	[74]
Rutin	SARS-CoV-2-3CLPro	N/A	31.26 μM	*Fagopyrum tataricum*	[74]
Vicenin-2	SARS-CoV-2-3CLPro	N/A	38.86 μM	*Citrus Reticulata*	[74]
**PLPro**					
Kazinol F	MERS-CoV-PLpro	N/A	39.5 µM	*Broussonetia papyrifera*	[72]
Broussochalcone A	MERS-CoV-PLpro	N/A	42.1 µM	*Broussonetia papyrifera*	[72]
Broussoflavan A	MERS-CoV-PLpro	N/A	49.1 µM	*Broussonetia papyrifera*	[72]
Isoliquiritigenin	MERS-CoV-PLpro	N/A	82.2 µM	*Broussonetia papyrifera*	[72]
Kazinol A	MERS-CoV-PLpro	N/A	88.5 µM	*Broussonetia papyrifera*	[72]
Kazinol B	MERS-CoV-PLpro	N/A	94.9 µM	*Broussonetia papyrifera*	[72]
Kazinol J	MERS-CoV-PLpro	N/A	55.0 µM	*Broussonetia papyrifera*	[72]
3′-(3-methylbut-2-enyl)-3′,4′,7-trihydroxyflavane	MERS-CoV-PLpro	N/A	48.8 µM	*Broussonetia papyrifera*	[72]
Cryptotanshinone	SARS-CoV-PLPro	N/A	0.8 µM	*Salvia miltiorrhiza*	[281]
Diplacone	SARS-CoV-PLPro	N/A	10.4 µM	*Paulownia tomentosa*	[284]
Broussochalcone A	SARS-CoV-PLPro	N/A	9.2 µM	*Broussonetia papyrifera*	[72]
Broussoflavan A	SARS-CoV-PLPro	N/A	30.4 µM	*Broussonetia papyrifera*	[72]
Chalcone	SARS-CoV-PLPro	Vero cells	1.2 µM	*Angelica keiskei*	[285]
Curcumin	SARS-CoV-PLPro	N/A	5.7 µM	*Curcuma longa*	[283]
Dihydrotanshinone I	SARS-CoV-PLPro	N/A	4.9 µM	*Salvia miltiorrhiza*	[281]
6-geranyl-4′,5,7-trihydroxy-3′,5′-dimethoxyflavanone	SARS-CoV-PLPro	N/A	13.9 µM	*Paulownia tomentosa*	[284]
Hirsutanonol 5	SARS-CoV-PLPro	N/A	7.8 µM	*Alnus japonica*	[283]
Hirsutenone 2	SARS-CoV-PLPro	N/A	4.1 µM	*Alnus japonica*	[283]
4-hydroxyisolonchocarpin	SARS-CoV-PLPro	N/A	35.4 µM	*Broussonetia papyrifera*	[72]
Isoliquiritigenin	SARS-CoV-PLPro	N/A	24.6 µM	*Broussonetia papyrifera*	[72]
Kaempferol	SARS-CoV-PLPro	N/A	16.3 µM	*Broussonetia papyrifera*	[72]
Kazinol A	SARS-CoV-PLPro	N/A	66.2 µM	*Broussonetia papyrifera*	[72]
Kazinol B	SARS-CoV-PLPro	N/A	31.4 µM	*Broussonetia papyrifera*	[72]
Kazinol F	SARS-CoV-PLPro	N/A	27.8 µM	*Broussonetia papyrifera*	[72]
Kazinol J	SARS-CoV-PLPro	N/A	15.2 µM	*Broussonetia papyrifera*	[72]
3′-(3-methylbut-2-enyl)-3′,4′,7-trihydroxyflavane	SARS-CoV-PLPro	N/A	35.8 µM	*Broussonetia papyrifera*	[72]
3′-O-methyldiplacol	SARS-CoV-PLPro	N/A	9.5 µM	*Paulownia tomentosa*	[284]
4′-O-methyldiplacol	SARS-CoV-PLPro	N/A	9.2 µM	*Paulownia tomentosa*	[284]
3′-O-methyldiplacone	SARS-CoV-PLPro	N/A	13.2 µM	*Paulownia tomentosa*	[284]
4′-O-methyldiplacone	SARS-CoV-PLPro	N/A	12.7 µM	*Paulownia tomentosa*	[284]
Mimulone	SARS-CoV-PLPro	N/A	14.4 µM	*Paulownia tomentosa*	[284]
Oregonin	SARS-CoV-PLPro	N/A	20.1 µM	*Alnus japonica*	[283]
Tanshinone IIA	SARS-CoV-PLPro	N/A	1.6 µM	*Salvia miltiorrhiza*	[281]
Tomentin A	SARS-CoV-PLPro	N/A	6.2 µM	*Paulownia tomentosa*	[284]
Tomentin B	SARS-CoV-PLPro	N/A	6.1 µM	*Paulownia tomentosa*	[284]
Tomentin C	SARS-CoV-PLPro	N/A	11.6 µM	*Paulownia tomentosa*	[284]
Tomentin D	SARS-CoV-PLPro	N/A	12.5 µM	*Paulownia tomentosa*	[284]
Tomentin E	SARS-CoV-PLPro	N/A	5.0 µM	*Paulownia tomentosa*	[284]
Rubranol	SARS-CoV-PLPro	N/A	12.3 µM	*Alnus japonica*	[283]
Rubranoside A	SARS-CoV-PLPro	N/A	9.1 µM	*Alnus japonica*	[283]
Rubranoside B	SARS-CoV-PLPro	N/A	8.0 µM	*Alnus japonica*	[283]
Papyriflavonol A	SARS-CoV PLPro	N/A	3.7 µM	*Broussonetia papyrifera*	[72]
Quercetin	SARS-CoV PLPro	N/A	8.6 µM	*Broussonetia papyrifera*	[72]
Quercetin-β-galactoside	SARS-CoV PLPro	N/A	51.9 µM	*Broussonetia papyrifera*	[72]
Broussochalcone B	SARS-CoV-PLPro	N/A	11.6 µM	*Broussonetia papyrifera*	[72]
**RdRp**					
Amentoflavone	SARS-CoV-2 RdRp	RD cells	13.17 µM	*Selaginella tamariscina*	[77]
Baicalein	SARS-CoV-2 RdRp	Vero CCL-81	4.5 µM	*Scutellaria baicalensis*	[69]
Baicalin	SARS-CoV-2 RdRp	Vero CCL-81	9 µM	*Scutellaria baicalensis*	[69]
Corilagin	SARS-CoV-2 RdRp	Vero CCL-81	0.13 µM	*Caesalpinia coriaria*	[286]
Luteolin	SARS-CoV-2 RdRp	N/A	4.6 µM	*Apium Graveolens*	[287]
Lycorine	MERS-CoV RdRp	Vero CCL-81	1.41 µM	*Lycoris Radiata*	[87]
Lycorine	SARS-CoV RdRp	Vero CCL-81	1.02 µM	*Lycoris Radiata*	[87]
Lycorine	SARS-CoV-2 RdRp	Vero CCL-81	0.88 µM	*Lycoris Radiata*	[87]
Quercetin	SARS-CoV-2 RdRp	N/A	6.9 µM	*Alium Cepa*	[287]
**SARS-CoV nsp13-Helicase/ATPase activity**					
Myricetin	nsp13 ATPase	N/A	2.71 µM	N/A	[78]
Scutellarein	nsp13 ATPase	N/A	0.86 µM	*Scutellaria baicalensis*	[78]
Baicalein	nsp13 ATPase	N/A	0.47 µM	*Scutellaria baicalensis*	[288]
Baicalein	nsp13 helicase	Vero E6	2.9 µM	*Scutellaria baicalensis*	[70]
Dihydro-myricetin	nsp13 helicase	Vero E6	25.6 µM	N/A	[70]
Diosmetin	nsp13 helicase	Vero E6	10.6 µM	*Vicia cracca*	[70]
Ellagic acid	nsp13 ATPase/helicase	N/A	2.8 µM	N/A	[83]
Flavanone	nsp13 helicase	Vero E6	0.52 µM	N/A	[70]
Flavanone-7-O-glucoside	nsp13 helicase	Vero E6	2.88 µM	N/A	[70]
(−)-Gallocatechin gallate	nsp13 helicase	N/A	1.34 µM	*Camellia sinensis*	[83]
Licoflavone C	nsp13 helicase	Vero E6	1.34 µM	*Genista ephedroides*	[70]
Kaempferol	nsp13 helicase	Vero E6	0.76 µM	N/A	[70]
Katacine	nsp13 helicase	N/A	5.98 µM	*Polygonum coriarium*	[83]
Licoflavone C	nsp13 ATPase (in the presence of BSA, TCEP and polyrA)	Vero E6	18.3 µM	*Genista ephedroides*	[70]
Linoleic acid	nsp13 helicase/ATPase	N/A	4.3 µM	N/A	[289]
Myricetin	nsp13 helicase	Vero E6	0.41 µM	N/A	[70]
Oleic acid	nsp13 helicase/ATPase	N/A	14 µM	N/A	[289]
Gossypol	nsp13 helicase/ATPase	N/A	1.3 µM	*Gossypium* spp.	[289]
Prunetin	nsp13 helicase	Vero E6	11.5 µM	*Prunus emarginata*	[70]
Punicalagin	nsp13 helicase	Vero cells, A549-ACE2	0.43 µM	*Punica granatum*	[83]
Quercetin	nsp13 helicase	Vero E6	0.53 µM	N/A	[70]
Rhodiosin	nsp13 helicase	N/A	0.48 µM	*Rhodiola* spp.	[83]
Rosmanol	nsp13 helicase	N/A	8.93 µM	*Rosmarinus officinalis* L.	[83]
Tannic acid	nsp13 helicase	N/A	1.25 µM	N/A	[83]
Wogonin	nsp13 helicase	Vero E6	24.9 µM	*Scutellaria baicalensis*	[70]
**Dengue virus**					
Sotetsuflavone	NS5 RdRp	N/A	0.16 µM	*Dacrydium araucarioides*	[107]
Apigenin	NS5 RdRp and restores STAT2 inhibition by NS5	IFN-I competent Huh7 cells, engineered K562 cell platform	EC_50_ 29.7 µM	N/A	[108]
Luteolin	NS5 RdRp and restores STAT2 inhibition by NS5	IFN-I competent Huh7 cells, engineered K562 cell platform	EC_50_ 9.2 µM	N/A	[108]
Bisdemethoxycurcumin	NS2B/NS3 protease (DENV2)	BHK-21 cells	36.23 µM	*Curcuma longa*	[110]
Curcumin	NS2B/NS3 protease (DENV2)	BHK-21 cells	66.0 µM	*Curcuma longa*	[110]
Myricetin	NS2B/NS3 protease	N/A	8.46 µM	N/A	[109]
**Influenza A**					
Apigenin	Neuraminidase	MDCK	33 µM	*Rhodiola rosea roots*	[123]
Astragalin	Neuraminidase	MDCK	38 µM	*Rhodiola rosea roots*	[123]
Cosmosiin	Neuraminidase	MDCK	47 µM	*Rhodiola rosea roots*	[123]
Demethoxymatteucinol	Neuraminidase	MDCK	30 µM	*Pentarhizidium orientale*	[125]
Gossypetin	Neuraminidase	MDCK	3 µM	*Rhodiola rosea roots*	[123]
Herbacetin	Neuraminidase	MDCK	9 µM	*Rhodiola rosea roots*	[123]
Hispidulin	Neuraminidase	MDCK	19.83 µM	*Salvia plebeia R. Br*	[124]
3′-hydroxy-5′-methoxy-6,8-dimethylhuazhongilexone	Neuraminidase	MDCK	24 µM	*Pentarhizidium orientale*	[125]
Kaempferol	Neuraminidase	MDCK	11 µM	*Rhodiola rosea roots*	[123]
Linocinamarin	Neuraminidase	MDCK	44 µM	*Rhodiola rosea roots*	[123]
Luteolin	Neuraminidase	MDCK	17.96 µM	*Salvia plebeia R. Br*	[124]
Matteucin	Neuraminidase	MDCK	24 µM	*Pentarhizidium orientale*	[125]
Matteucinol	Neuraminidase	MDCK	25 µM	*Pentarhizidium orientale*	[125]
Methoxymatteucin	Neuraminidase	MDCK	25 µM	*Pentarhizidium orientale*	[125]
Nicotiflorin	Neuraminidase	MDCK	32 µM	*Rhodiola rosea roots*	[123]
Quercetin	Neuraminidase	MDCK	2 µM	*Rhodiola rosea roots*	[123]
Rosmarinic acid methyl ester	Neuraminidase	MDCK	16.65 µM	*Salvia plebeia R. Br*	[124]
Rutin	Neuraminidase	MDCK	34 µM	*Rhodiola rosea roots*	[123]
Rhodiolinin	Neuraminidase	MDCK	10 µM	*Rhodiola rosea roots*	[123]
Rhodionin	Neuraminidase	MDCK	32 µM	*Rhodiola rosea roots*	[123]
Rhodiosin	Neuraminidase	MDCK	57 µM	*Rhodiola rosea roots*	[123]
Nepetin	Neuraminidase	MDCK	11.18 µM	*Salvia plebeia R. Br*	[124]
2′,4′dihydroxy-6′-methoxy-3′,5′-dimethylchalcone	Neuraminidase	HEK293, MDCK	8.23 µM (H1N1) 5.07 µM (H9N2) 7.02 μM (H1N1 WT) 8.84 μM (H1N1-H274Y mutation)	*Cleistocalyx operculatus*	[126]
Myricetin-3′,5′-dimethylether-3-O-β-D-galactopyranoside	Neuraminidase	HEK293, MDCK	8.86 µM (H1N1) 6.50 µM (H9N2) 7.10 μM (H1N1 WT) 9.34 μM (H1N1-H274Y mutation)	*Cleistocalyx operculatus*	[126]
Berberine	HAE cells, blocks nuclear export of IAV ribonucleoprotein to cytoplasm	MDCK, A549, LET1, HAE	16 µM	*Berberis* sp.	[129]
**HIV-1**					
Oleanolic acid	Protease	N/A	10 µg/mL	*Xanthoceras sorbifolia*	[290]
3-oxotirucalla-7, 24-dien-21-oic acid	Protease	N/A	20 µg/mL	*Xanthoceras sorbifolia*	[290]
Apigenin	Integrase	N/A	22 µM	*Punica granatum*	[94]
Ellagic acid	Integrase	N/A	0.075 µM	*Punica granatum*	[94]
Betulinic acid	Integrase	N/A	96.5 µM	*Punica granatum*	[94]
Kuwanon-L	RT-associated RDDP	TZM-bl (modified HeLa)	0.99 µM	*Xanthocer assorbifolia*	[291]
Kuwanon-L	RT-associated RNase	TZM-bl (modified HeLa)	0.57 µM	*Xanthocer assorbifolia*	[291]
Luteolin	Integrase	N/A	6.5 µM	*Punica granatum*	[94]
Luteolin 7-*O*-glucoside	Integrase	N/A	8.5 µM	*Punica granatum*	[94]
Punicalins	Integrase	N/A	0.09 µM	*Punica granatum*	[94]
Punicalagins	Integrase	N/A	0.065 µM	*Punica granatum*	[94]
Corilagin	Reverse transcriptase	MT4 T-lymphoid, MAGI cells	9.3 µM	*Phyllanthus amarus*	[93]
Norisoboldine	Reverse transcriptase	N/A	153.7 μg/mL	*Croton echinocarpus*	[292]
L-chicoric acid	Reverse transcriptase (presence of heteropolymeric template)	MT-2	17 µM	*Echinacea purpurea*	[92]
Geraniin	Reverse transcriptase	MT4 T-lymphoid, MAGI cells	1.9 µM	*Phyllanthus amarus*	[93]
1-methoxyoxalyl-3,5-DCQA	Reverse transcriptase (presence of heteropolymeric template)	MT-2	7 µM	*Echinacea* spp.	[92]
Apigenin	RT-associated RNase	N/A	16.1 µM	*Punica granatum*	[94]
Betulinic acid	RT-associated RNase	N/A	2.0 µM	*Punica granatum*	[94]
Ellagic acid	RT-associated RNase	N/A	1.4 µM	*Punica granatum*	[94]
Luteolin	RT-associated RNase	N/A	3.7 µM	*Punica granatum*	[94]
Oleanolic acid	RT-associated RNase	N/A	6.7 µM	*Punica granatum*	[94]
Punicalins	RT-associated RNase	N/A	0.18 µM	*Punica granatum*	[94]
Punicalagins	RT-associated RNase	N/A	0.12 µM	*Punica granatum*	[94]
Ursolic acid	RT-associated RNase	N/A	5.7 µM	*Punica granatum*	[94]
3-O-(3′,3′-dimethylsuccinyl) betulinic acid—Bevirimat	p25-to-p24 conversion	PBMC, MT-2	10.3 nM	*Syzygium claviflorum*	[95]
**Zika virus**					
Astragalin	NS2B-NS3Pro	N/A	112.0 µM	*Phytolacca americana*	[101]
Epicatechin gallate	NS2B-NS3Pro	N/A	98.0 µM	*Camellia sinensis*	[101]
Epigallocatechin gallate	NS2B-NS3Pro	N/A	87.0 µM	*Camellia sinensis*	[101]
Gallocatechin gallate	NS2B-NS3Pro	N/A	99.0 µM	*Camellia sinensis*	[101]
Luteolin	NS2B-NS3Pro	N/A	53.0 µM	N/A	[101]
Myricetin	NS2B-NS3Pro	N/A	22.0 µM	N/A	[101]
Rutin	NS2B-NS3Pro	N/A	112.0 µM	N/A	[101]
(−)-epigallocatechin-3-gallate	NS3 helicase–ATPase activity	N/A	2.95 µM	*Camellia sinensis*	[102]
**Chikungunya virus**					
Berberine	Host MAPK pathway	HEK293T	4.5 µM	*Anamirta cocculus*	[115]
Harringtonine	Nsp2 protease	BHK-21	0.24 µM	*Cephalotaxus harringtonia*	[114]
Tomatidine	Nsp2 protease	Huh7	1.3 µM	*Solanum dulcamara*	[293]
Apigenin	Replicase complex	BHK-21	70.8 µM	N/A	[113]
Chrysin	Replicase complex	BHK-21	126.6 µM	N/A	[113]
Naringenin	Replicase complex	BHK-21	118.4 µM	N/A	[113]
Silybin	Replicase complex	BHK-21	92.3 µM	N/A	[113]
Withaferin A	Nsp2 protease	BHK-21	0.51 µM	*Withania somnifera*	[294]
Prostratin	Not established/replication machinery	Vero cells, BGM, HEL	8 µM (Vero) 7.6 µM (BGM) 7.1 µM (HEL)	*Trigonostemon howii*	[116]
Baicalein	Replicase complex	Vero cells	7 µM	*Scutellaria baicalensis*	[48]
Fisetin	Replicase complex	Vero cells	29.5 µM	N/A	[48]
Quercetagetin	Replicase complex	Vero cells	43.52 µM	N/A	[48]
Silymarin	Replicase complex	Vero cells	16.9 µg/mL	*Silybum marianum*	[117]
Trigocherrierin A	CHIKV-induced cell death	Vero cells	0.6 µM	*Trigonostemon cherrieri*	[295]
